# Is HPS a valuable component of a STEM education? An empirical study of student interest in HPS courses within an undergraduate science curriculum

**DOI:** 10.1007/s13194-021-00433-x

**Published:** 2022-02-26

**Authors:** Greg Lusk

**Affiliations:** 1 Lyman Briggs College and the Department of Philosophy, Michigan State University, East Lansing, MI USA; 2 Present Address: Department of Philosophy, Durham University, Durham, UK

**Keywords:** Philosophy of science, Science education, STEM education, Humanities and science, HPS, Science studies, Interdisciplinarity

## Abstract

This paper presents the results of a survey of students majoring in STEM fields whose education contained a significant history, philosophy and sociology (HPS) of science component. The survey was administered to students in a North American public 4-year university just prior to completing their HPS sequence. The survey assessed students’ attitudes towards HPS to gauge how those attitudes changed over the course of their college careers, and to identify the benefits and obstacles to studying HPS as a component of their STEM education. The survey reveals that students generally found unexpected value in taking HPS within their STEM curriculum. It also reveals that framing HPS courses as a means of gaining communication skills necessary to be an influential scientist seems to resonate with students. However, students also identified several factors limiting engagement with HPS content, including the length and density of required readings and assessment via essays and papers.

Writing in the *PSA Proceedings* in 1974, Michael Martin lamented “a great deal has been written on the philosophy of science; perhaps even more has been written in science education. However, surprisingly little has been written on the relation between the two areas” (Martin, 1974, 293). Despite Martin’s subsequent writings on the subject, this statement might still be true today. However, there seems to be a burgeoning interest in the value of philosophy of science – and other science studies disciplines like history and sociology – when educating scientists, particularly at the university level. In a widely shared piece on *Aeon*, Subrena Smith argues that philosophy of science can play an important role in university-level science education and should not be made subservient to the sciences (Smith, 2017). Grüne-Yanoff (2014) articulates several benefits that philosophy of science can bring to science training to help create better scientists and suggests altered teaching approaches to realize those benefits more quickly. Even more recently, the prominent history of science journal *ISIS* included a focus section on pedagogy (see Rader, 2020). Though not primarily targeting a higher-ed audience, the journal *Science Education* has had a Science Studies and Science Education section since 2008 (see Duschl et al., 2008 and articles therein and since).

Within these articles, it is not uncommon to find analyses of obstacles that arise when attempting to engage STEM students in courses that reflect upon science. Within the *ISIS* focus section, Vivien Hamilton, a historian of science, and Daniel M. Stoebel, a biologist, reflect on concerns that what scientists’ see as helpful analyses of historical episodes for training students may be at odds with the nuanced and non-judgmental narratives historians of science are comfortable providing (Hamilton & Stoebel, 2020). Often however, many obstacles are seen as arising from STEM students themselves: there is a perception that STEM students are resistant to non-STEM courses in their curriculum. Smith cites episodes where students question her authority in the classroom and remarks that “students are doubtful that philosophers have anything useful to say about science” (Smith, 2017). Similarly, Till Grüne-Yanoff (2014) paints a picture of science students as disinterested in, and irked by, mandatory non-science breadth requirements, including philosophy of science. He claims university science students not only lack knowledge about how to reflect upon science, but they have little grasp of science itself.

STEM student resistance to such courses may have social and educational consequences. For example, there is recent evidence that medical students trained in the humanities or interpretive social sciences display greater levels of empathy compared to those receiving a positivist STEM education, empathy which in turn makes them more effective with their patients (Olsen & Gebremariam, 2020). Additional study is required, but it is reasonable to suggest resistance to this training might diminish empathy, if it is indeed learned. Furthermore, student engagement is widely accepted as an important influence on student learning, especially among the lowest-ability students (Carini et al., 2006; Kahu, 2013; Trowler, 2010).

Yet, outside of personal anecdotes, there is little data available to suggest that STEM students are or are not resistant to classes that reflect upon science. And even if they are resistant, is that resistance overcome with experience? Do students end up finding value in courses that encourage them to reflect upon science? The way these courses should be taught to undergrads, and how effective they might be, depends on the answers to these questions.

This contribution attempts to answer the above questions empirically. It presents results of a survey of fifty-two students majoring in STEM fields whose education contained a significant history, philosophy and sociology (HPS) of science component. The survey was administered to students in a North American public 4-year University just prior to completing their HPS sequence. The survey asked students about their attitudes towards HPS, about how those attitudes changed over the course of their college careers, and about the benefits and obstacles to studying HPS as a component of their STEM education. The survey reveals that students generally found HPS to be a valuable addition to their STEM curriculum. It also reveals that framing HPS courses as a means of gaining the communication skills necessary to be an influential scientist seems to resonate with students. However, students also identified several factors limiting engagement with HPS content, including the length and density of required readings and assessment via essays and papers. This research suggests ways to positively alter student experiences of HPS to more quickly overcome student resistance and enhance learning. Overall, the value that students find in HPS courses helps to justify the inclusion of HPS as part of a STEM curriculum.

## Survey background: HPS within a STEM curriculum

To assess non-major student interest and student-perceived value of HPS courses, the HPS Experience Survey was created to identify STEM students’ beliefs, motivations, and aspirations regarding HPS courses. The survey was administered in Fall 2020 at Michigan State University– a four-year public university located in the Midwest region of the United States. Students taking the survey were enrolled in Lyman Briggs College (henceforth “LBC”) one of several undergraduate “residential colleges” within the larger University and the only one that admits solely STEM students. As such, students must major in a STEM field (or in HPS) to graduate from LBC.

The unique setup of LBC and the way it incorporates HPS into a STEM curriculum are factors that deeply influence student experiences of HPS and possible interpretations of the survey. Admission into LBC is optional and requires no prerequisites beyond acceptance into the University and a desire to major in a STEM field (which includes HPS). LBC admits about 600 students each year from the University. This means that students self-select into, and can self-select out of, LBC. The perception on campus is that LBC is an honors college for would-be medical doctors. In actuality, not all, or sometimes even a majority, of LBC’s students are members of the University’s Honors College. However, students do often major in the biomedical sciences. For example, in 2018 there were 33 different majors declared by students in LBC. Of those majors, Human Biology (282), Neuroscience (202), and Physiology (102) were the most popular, and the only other majors with more than 50 students were Genomics & Molecular Genetics and Biochemistry.

Beyond its small size, admission into LBC uniquely affords students access to certain introductory STEM courses and the HPS sequence. LBC’s students are given exclusive access to smaller-enrollment introductory science courses taught by college faculty (in Math, Chemistry, Biology, and Physics). These courses often emphasize experiential learning more than their counterparts offered outside LBC and are perceived by students to be more difficult. Since only introductory-level science courses are taught in LBC, students complete their more advanced STEM courses in the relevant department within the broader university, but they return for their HPS courses.

Students are also afforded the ability to fulfill some of their university distribution requirements through LBC’s HPS sequence. The HPS sequence typically consists of four courses that replace the university mandated introductory humanities and social science courses while also fulfilling the university’s writing requirement. The first course is an introduction to history, philosophy, and sociology of science, taken in a student’s first year (unless exempted due to advanced placement credits). Enrollment in these courses is typically capped at 24. The second and third courses are taken in the third year of study, after students complete their science courses in LBC. These third-year courses cover a variety of different themes, for example “Science and the Public”, “Science and the Environment” or “Science of Sex and Gender.” The enrollment in these courses is typically capped at 30. The final course in the sequence is a capstone course, designed to be taken in a student’s last year of study. These capstone courses of 15 students often have narrow topics but ask the students to reflect and utilize the knowledge they have gained across their collegiate career.

It is important to note that – at least after the first year - there is a wide variety of course offerings, and thus, one should not expect students completing the sequence to be exposed to the same content. The courses do, however, have a similar emphases and overall curricular goals. Broadly, the courses aim to help students becomes scientists that are not only competent in the lab or field but can also recognize the social ramifications of their work and can communicate those ramifications to non-scientists. Thus, one of the central goals of the HPS sequence is to ensure that students acquire successful strategies for effective research, writing, and self-expression. Extensive writing instruction and practice are built into the HPS sequence. The forms of writing may vary but include traditional essays (with opportunities to edit drafts with peers and revise after consultation with an instructor), keeping a journal of personal experiences with scientific issues, or even writing podcast scripts and digitally recording them.

At the same time, these HPS courses challenge students to consider the rational and cultural forces that affect the practice of science. It is typical for faculty to develop experiential learning activities to achieve this aim. For example, students in the introductory course may examine the question “What is science?” and explore the demarcation problem. To do so, students engage in a “black box” activity (based on Hardcastle & Slater, 2014) where students investigate the contents of a box that they cannot open. Students must create the rules for the investigation (e.g. what constitutes opening the box, what methods of investigation are permissible), determine whether they should be able to work together (and how), and discuss how their chosen rules for investigation would impact other scientific practices. The third year “Science and the Public” course has utilized an activity (see Charenko & Louson, 2019) where students role played different stakeholders responding to an environmental disaster reminiscent of the fallout of Chernobyl in Britain. One capstone course (pre-COVID) employed the boardgame Pandemic: Legacy to imagine a world facing a global pandemic. The class explored the ethics of triage, the role of institutions in protecting health, and public trust in vaccines, among other topics.^1^

While the HPS sequence is often presented to students as enhancing science communication, it is not the only way these courses are described within LBC. LBC sometimes frames these courses as ethics courses – where students learn to be good scientists – or frames them as critical thinking courses.

## Survey methodology: Assessing student engagement in HPS courses

The HPS experience survey was made available to students within LBC at the beginning of their capstone courses in the HPS sequence in Fall of 2020. A link to the optional survey was sent to members of each course section at the discretion of their instructor. Courses in Fall 2020 were fully online due to COVID-19 restrictions, though the experiences that the students reflect upon were almost entirely traditional in-person interactions. There is no evidence that COVID-19 restrictions significantly altered results.

The survey was anonymous, optional, and no personal identifiable information was gathered. Students were informed before consenting to the survey that results might be used to help students design projects in one section of the capstone course, be used in other forms of college analysis, or for purposes and research beyond LBC. The results were kept by the survey creator and not shared among instructors. The University’s Institutional Review Board Office judged that this research did not require IRB approval and that it complies with the relevant federal regulations beyond those under the purview of the IRB.

**Fig. 1:**
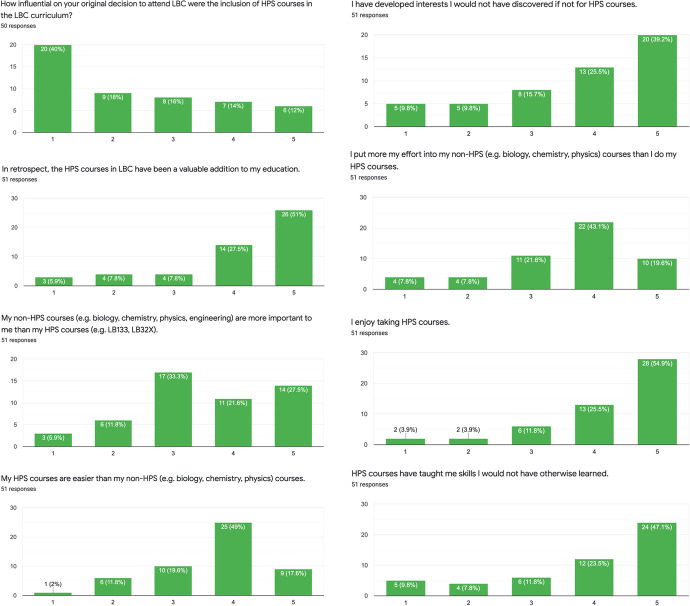
Strongly Disagree (or not at all influential in the case of the first question) is 1, whereas strongly agree (or extremely influential in the case of the first question) is 5

The survey consisted of three sections: a demographic section, a section asking about overall student experience in LBC, and a section addressing experiences in HPS classes. The HPS section of the survey, which will be the focus here, asked three types of questions: (1) multiple choice questions on student educational background, (2) 5-point Likert scale questions regarding the student’s perception of LBC, its HPS courses, and the influence of such courses on the student and (3) open ended response questions regarding student experience in LBC and its HPS courses. Open-ended responses were coded following an inductive approach in order to analyze students’ perceptions regarding: 1) what was valuable; 2) skills gained; and 3) obstacles presented in HPS courses. The HPS section of survey is provided in Figure 1 and Table 1 in the appendix.

In Fall 2020 there were 165 students enrolled across 12 sections of the capstone course; 52 members of this group responded to the survey with 36 students providing demographic information. The demographic information revealed that 88.6% (31) of respondents had completed three years of university education, 8.6% (3) had completed four years, and 2.9% (1) had completed one year (35 rather than 36 students responded to this question). The HPS section of the survey received 51 responses for each Likert-scale question. Of those responding, 49% (25) students had taken three HPS courses at the time of the survey, 31.4% (16) had taken two HPS courses, 11.8% (6) four, 3.9% (2) one, and five and more than six courses were each selected by 2.8% (1) of respondents. Those indicating they took less than three courses were likely exempt from the introductory HPS course due to university credit earned in high school (usually through advanced placement English classes) or were taking the fourth course in the sequence early or at the same time as the capstone due to scheduling conflicts. While 98% (50) of those surveyed were not HPS majors, one individual was, and this is likely responsible for the single >6 response. Though the focus in this paper is on students who are STEM majors, this response was not removed from the sample because it is possible that this respondent is a double major in a STEM field in addition to HPS and it is unlikely that their original intention in joining LBC was to major in HPS.

## Survey results: Lessons from the HPS experience survey

### Surveyed students find the inclusion of HPS courses in a STEM curriculum to be a valuable addition to their education, but HPS courses were not a deciding factor in their choice of program

When asked whether, in retrospect, students viewed the inclusion of HPS courses in their curriculum as valuable additions, a majority (51%) of those surveyed strongly agree, with 78.5% agreeing or strongly agreeing. However, the inclusion of the HPS sequence did not typically motivate students to join LBC. The most common response when asked if the inclusion of HPS courses influenced student decisions to attend LBC was “not at all influential” (40%). On the non-HPS section of the survey, students repeatedly explained that their motivation to attend LBC came from access to a more challenging science curriculum with smaller class sizes. That arts courses are offered alongside science courses is mentioned only sparingly. For example, though one respondent claimed “A college that represented a mixed focus on both the humanities and science seemed like exactly the thing I’d enjoy” and another “I wanted a better understanding of how science came to be how it is today,” there were far more offering some variant of “I like the concept of the smaller classroom sizes and I heard the teaching and academics are superior to the rest of the school.”

That HPS – one of the unique characteristics of LBC – is infrequently a factor motivating enrollment might indicate that students come to see the value in HPS over time. That is, students are initially neutral towards HPS courses, do not know what they are, or find HPS courses to be a burden or unnecessary to their education. Evidence for this interpretation is given in the open-ended responses. Students repeatedly advise future students to be open minded about HPS, which may reveal that students were initially not open minded and resistant to these courses. For example, one student writes “Go in with an open mind and find out stuff about your opinions and ideology you never knew was there” while another claims “HPS is not as big of a hassle as it seems.” Some students mention HPS courses when asked how LBC exceeded their expectations. One student wrote, “I didn’t come into LBC with expectations about the integration of science and the humanities (I thought it was a cool feature, but didn’t care too much about it), but ended up loving HPS classes and the way they’ve broadened my worldview.” While students may not have initially perceived HPS courses as meaningful additions to their educations, after having experiences with these courses, they grow to like them. In fact, a few students directly reference a changed perception of HPS; for example, “My feelings for LBC have not changed very much since Freshman year, besides developing a greater appreciation for HPS.” There is little indication that if resistance was present, that it persists.

### Surveyed students enjoyed taking HPS courses and they indicate that they gained skills and interests that they otherwise would not have

Results of the survey clearly indicate that a majority of students surveyed enjoyed taking HPS courses (54.9% strongly agree, 25.5% agree). A majority of students also indicated that they had developed interests or skills that they otherwise would not have (47.1% strongly agree, 23.5% agree). The skills that students mention gaining vary. For example, communication skills (including “communication”, “reading”, “writing”, and “listening”) were frequently mentioned, especially when students were asked to share one specific skill they learned. As one person commented, they learned “Communication and how to speak up when I have an opinion”, while other students wrote “The ability to quickly skim through a text while retaining the most valuable information” and “how to write research papers.” Many answers combine various elements of the framings – communication, critical thinking, and ethics – for HPS employed by LBC. One student learned to “Critically understand the author’s argument” while another learned “how to write an argumentative ethics paper.”

It is tempting to draw the conclusion that the major benefit to students of HPS courses are in fact enhanced communication skills. However, in this case one should be cautious: LBC frequently frames HPS as an opportunity to develop the communication skills needed to become a well-rounded scientist. An epistemically safer claim would be that framing the value of HPS in terms of enhanced communication can successfully resonate with STEM students.

Students see other benefits as well. Across the open answer questions, the opportunity for personal growth and the gaining of perspective are often mentioned as what other students should know about HPS, indicating that these skills are highly valued. Several students mention that HPS provided “eye opening” experiences while others comment that they got to “hear a lot of different perspectives.” This desire to widen one’s perspective seems to be echoed in the suggested additions to the HPS curriculum. Frequently mentioned topics included systematic racism, social (in)justice, mental health, and gender equality. These topics are somewhat unsurprising suggestions, as Black Lives Matter protests and the #MeToo movement were receiving significant attention around the time of the survey. Still, these suggestions may signal a desire for STEM students to examine the role of their chosen disciplines in present day social issues. It seems that STEM students want to become “well-rounded” scientists.

### Surveyed students generally felt that their HPS courses were easier than their non-HPS courses, of equal or lesser importance, and they put more effort into their non-HPS courses

Studies in higher education tend to link engagement with positive learning experiences (Carini et al., 2006; Kahu, 2013; Trowler, 2010). In that light, the responses comparing HPS to non-HPS courses are encouraging. The survey suggests that HPS and non-HPS courses may be on par, or at least, STEM students are unsure of how to gauge their relative importance: students most frequently select neither agree or disagree (33%) when asked whether non-HPS courses are more important than HPS courses. More students agree or strongly agree (49.1% combined) than disagree that their non-HPS courses are more important, but that is expected given that they major in STEM. The same reasoning can be applied to students’ effort in such courses: students indicate they are more likely to put more effort into their non-HPS courses (41.3% agree, 19.6% strongly agree). One would expect this, especially since a majority of students indicate that they find HPS courses slightly easier (49% agree that HPS courses are easier, 19.6% strongly agree). LBC draws distinctions between HPS and STEM courses, with the HPS faculty forming its own disciplinary group. It would be interesting to see if abolishing the distinction between HPS courses and science courses would result in changes of opinion (as Smith, 2017 seems to suggest). It also cannot be ruled out that the perceived easiness of HPS is linked to students’ favorable perceptions of them, a correlation known to plague student evaluations of teaching. This influence seems somewhat unlikely, given that the students in the survey have had a mix of different HPS professors and still report the classes being valuable.

### Obstacles for STEM student engagement in HPS courses include reading and writing assignments and a mismatch between course topics and student interest

One potential obstacle to engagement is assigning to STEM students the kind of reading and writing assignments that are often found in humanities courses. Several of the students who found HPS classes hard or not valuable mentioned the high demands on their reading skills.^2^ At least one student who indicated that their HPS courses had little value suggested that the assigned readings were “not needed.” Other students warned future students about the “dense” readings, while another commented that some readings led to “no engagement at all because the assigned reading wasn’t interesting/relevant for science majors. It turned more into an English class because the professor talked more about rhetoric and symbolism.”

Anecdotally, my experience suggests that STEM students in an HPS class often struggle to read texts that would be standard reading in a philosophy class of the same (or even lower) level. Clearly, one way to avoid this obstacle is to carefully choose readings. One might also rely on other kinds of media; many in LBC regularly assign podcasts or movies in lieu of a written text. At least one student approved of this approach: “I really enjoyed the use of multi-media (podcasts, books, etc.) in my previous courses.” STEM students might also be somewhat inclined to discount their writing abilities. At least one student connected this fear of writing to their performance in HPS: “I think that the HPS classes were hard for me because I really don’t like participating in class and I am an awful writer. So I always struggled when having to take the class which made me hate the classes because I wouldn’t do the best in them. I had a little anxiety when I had to go to the class.” In my experience, this sentiment about writing (and participation) is often mentioned by STEM students.

In addition to the reading and writing assignments, another obstacle is alignment between course topics and student interests. Since students may select three of the four classes in the sequence from many different course offerings, there is significant variability in student experience. This made drawing conclusions from open-ended questions about specific course topics difficult. However, one apparent theme is that a mismatch between student interests and the content of the course can be an obstacle to engagement. For example, one student who was not interested in becoming a doctor despised how “every course has a medicine section somewhere and frankly I could not care less about it at this point.” Another student studying biology was “hugely disappointed that my SENIOR SEMINAR for my BIOLOGY degree is a physics/theater/feminism discussion with hours of work outside of class that doesn’t relate to my degree…”. Others commented that they were unable to disagree with the professor or be uninterested in a topic, with one saying they felt pressure to write what the professor wanted on their essay. While such responses are merely suggestive, if one were designing an HPS curriculum for STEM students, the topical relevance of the content is an issue that deserves considerable attention. If HPS courses were targeting an audience with a diversity of scientific interests, it might suggest that courses adopt a survey approach rather than concentrate on specific topics.

## Survey limitations

There are certain limitations to this research that should be noted. The number of respondents is small, which limits the power with which conclusions can be drawn. The variety of courses on offer is large, and thus, the HPS content students have encountered may vary substantially. This makes drawing inferences about which content is effective for overcoming resistance, or what content should be changed, very difficult. The dependability of the survey questions is also unknown, as limited access to the population of interest has prevented pilot tests and validation. The use of 5-point Likert scale questions brings with it some ambiguity, for example, the middle “neither agree nor disagree” option could indicate that the respondent is neutral, does not know, or agrees with a strength of three out of five.

The survey population is also somewhat atypical, in part because they self-select into a college that has an HPS sequence and whose classes are perceived as more difficult than those standardly offered by the university. It should be noted that because students self-select into LBC, they can also leave LBC at any time and continue with the same major at the university. It is possible the HPS Experience Survey systematically fails to capture the opinions of students with significantly unfavorable views of HPS courses, because they left LBC before the capstone course where the survey took place. While this is possible, there is not yet evidence to support it. Though merely conjecture, I have not heard of a student leaving because they found HPS to be a burden, and in my experience, the students who excelled in HPS left LBC before the capstone, often to pursue an interest they discovered in their HPS courses within an unsupported major.^3^

Lastly, since a vast majority of the respondents to the HPS Experience Survey have completed two or more HPS courses (with the majority completing three or more), it cannot be established that a single class is sufficient to overcome resistance in reluctant students. That students may require time to realize the value of HPS could raise difficulties when integrating HPS into a STEM curriculum if the number of HPS courses is low or restricted to a single course.

## Conclusion: Overcoming resistance and turning obstacles into advantages

There is a perception that STEM students are disinterested in HPS and believe the subject has little to offer them. The HPS Experience Survey assessed whether this perception is accurate, and found it is not. After taking HPS courses, students retrospectively perceive value in them; they generally indicate that they learned skills that they would otherwise not have. In fact, the most agreed upon statement among students in the survey is “I enjoy taking HPS classes.” The HPS Experience Survey suggests that if STEM students were initially resistant, that resistance is dropped after experiences in HPS courses.

At the same time, this analysis offers recommendations for overcoming obstacles to STEM student engagement. STEM students were often dissatisfied with high reading demands in HPS courses. The learning objectives in an HPS course for non-majors may legitimately differ from those for HPS majors, and thus, efforts should be made to assign texts that advance these goals and match student abilities. One should not assume, for example, that a class designed for HPS majors would be engaging or accessible to STEM students. Instructors should consider assigning popular articles, videos, and podcasts – all of which may be more accessible for STEM students – where appropriate. Aligning the subject of this material to student interests is also likely to enhance engagement. Ensuring that the class material is accessible to STEM students – and aligns with their interests – is likely to be helpful when overcoming resistance.

That STEM students perceive value in HPS courses helps justify offering them within a STEM curriculum. Furthermore, what may initially seem like an obstacle – students being only interested in STEM – can be turned into an advantage. Student interest in STEM may be used to lower resistance in HPS courses that are required elements of a STEM curriculum. STEM students might initially be skeptical of the value of HPS courses because they perceive them as humanities or social science courses and are forced to take them to fulfill university breadth requirements. However, if STEM students do have a strong preference for classes that engage with science, then it is likely that they would prefer fulfilling such requirements through HPS courses rather than humanities or social science courses that are unrelated to their interests. One might hypothesize that faced with this choice, students would respond well to HPS courses and engage with them relatively deeply while still acquiring the important skills and knowledge that such requirements typically provide. The skills and knowledge may even be more directly applicable to student interests. Integrating HPS as a replacement for non-science related university requirements may somewhat undermine the purpose of a “breadth” requirement, but to many administrators, it may seem like a win-win when proposing the inclusion of HPS in STEM curricula.

## Appendix

The responses to the Likert-scale questions are summed up in Figure 1 below. In this table, a 1 is strongly disagree and 5 is strongly agree for all questions except the first, where 1 represents “not at all influential” and 5 “extremely influential.” Table 1 lists the open-ended questions.

**Table 1:** Open-ended questions asked on the HPS experience survey

1. Please share one specific skill [you would not have otherwise learned if not for HPS]. (33 Responses)	
2. What is your MOST memorable experience in an HPS course? Provide as much detail as you are comfortable. (50 Responses)	
3. What is your LEAST memorable experience in an HPS course? Provide as much detail as you are comfortable. ^a^(47 Responses)	
4. What TOPIC in an HPS course has been the MOST interesting to you, and why? (49 Responses)	
5. What TOPIC in an HPS course has been the LEAST interesting to you, and why? (46 Responses)	
6. What HPS related TOPIC should be taught in HPS classes but is not? (36 Responses)	
7. What HPS related SKILL should be taught in HPS classes but is not? (40 Responses)	
8. What is ONE thing future students should experience in their HPS classes? (45 Responses)	
9. What is the ONE thing future students should know about taking HPS classes? (49 Responses)	

^a^Several students pointed out that this was a poorly worded question: if it was not memorable, they wouldn’t remember it

## Footnotes

1 See Bursten and Strandmark (2021), as well as other contributions to this topical collection, for more engaged strategies for teaching HPS to STEM students.

2 Those reporting that the classes were hard relative to their science classes and those reporting that the classes were not valuable did not substantially overlap.

3 Some professors report that students cited HPS courses as a reason for leaving LBC when the HPS courses were additional mandatory requirements beyond their university distribution requirements.
